# Clinical and biological impact of SAMHD1 expression in mantle cell lymphoma

**DOI:** 10.1007/s00428-021-03228-w

**Published:** 2021-11-04

**Authors:** Magali Merrien, Agata M. Wasik, Elin Ljung, Mohammad H. A. Morsy, Joana de Matos Rodrigues, Mattias Carlsten, Georgios Z. Rassidakis, Birger Christensson, Arne Kolstad, Mats Jerkeman, Sara Ek, Nikolas Herold, Björn E. Wahlin, Birgitta Sander

**Affiliations:** 1 Department of Laboratory Medicine, Div. of Pathology, Karolinska Institutet and Karolinska University Hospital, SE14186, Stockholm, Sweden; 2 Department of Pathology, Karolinska University Hospital, Solna, Sweden; 3 Department of Immunotechnology, Lund University, Lund, Sweden; 4 PO Haematology and Unit of Haematology, Department of Medicine at Huddinge, Karolinska University Hospital and Karolinska Institutet, Solna, Sweden; 5 Center for Hematology and Regenerative Medicine, Department of Medicine Huddinge, Karolinska Institutet, Solna, Sweden; 6 Department of Oncology-Pathology, Karolinska Institutet, Stockholm, Sweden; 7 Department of Hematopathology, The University of Texas MD Anderson Cancer Center, Houston, TX USA; 8 Department of Oncology, Oslo University Hospital Radiumhospitalet, Oslo, Norway; 9 Department of Oncology, Lund University, Lund, Sweden; 10 Childhood Cancer Research Unit, Department of Women’s and Children’s Health, Karolinska Institutet, Solna, Sweden; 11 Paediatric Oncology, Astrid Lindgren Children’s Hospital, Karolinska University Hospital Solna, Solna, Sweden

**Keywords:** Mantle cell lymphoma, SAMHD1, Cytarabine, Prognostic factors

## Abstract

SAMHD1 is a deoxynucleoside triphosphate triphosphohydrolase (dNTPase) that restricts viral replication in infected cells and limits the sensitivity to cytarabine by hydrolysing its active metabolite, as recently shown in acute myeloid leukemia. Cytarabine is an essential component in the Nordic mantle cell lymphoma protocols (MCL2 and MCL3) for induction and high-dose chemotherapy treatment before autologous stem cell transplantation for younger patients with mantle cell lymphoma (MCL). We here investigated the expression of SAMHD1 in a population-based cohort of MCL (*N* = 150). SAMHD1 was highly variably expressed in MCL (range, 0.4% to 100% of positive tumor cells). Cases with blastoid/pleomorphic morphology had higher SAMHD1 expression (*P* = 0.028) and SAMHD1 was also correlated to tumor cell proliferation (*P* = 0.016). SAMHD1 expression showed moderate correlation to the expression of the transcriptional regulator SOX11 (*P* = 0.036) but genetic silencing of SOX11 and SAMHD1 by siRNA in MCL cell lines did not suggest mutual regulation. We hypothesized that expression of SAMHD1 could predict short time to progression in patients treated with Cytarabine as part of high-dose chemotherapy. Despite the correlation with known biological adverse prognostic factors, neither low or high SAMHD1 expression correlated to PFS or OS in patients treated according to the Nordic MCL2 or MCL3 protocols (*N* = 158).

**Supplementary Information:**

The online version contains supplementary material available at 10.1007/s00428-021-03228-w.

## Introduction

Mantle cell lymphoma (MCL) accounts for 5–8% of all non-Hodgkin lymphomas and previously entailed a dismal prognosis with a reported median overall survival (OS) of only 3–5 years [1–3]. Most cases express cyclin D1 as a result of the t(11;14)(q13;q33) *CCND1/IGH* translocation. Approximately 10% of MCL present with large cell morphology (blastoid or pleomorphic variants) and are often characterized by high tumor cell proliferation and may harbor *TP53* mutations. However, MCL with classical morphology can also show high proliferation and *TP53* aberrations and, in many studies, high tumor-cell proliferation and *TP53* aberrations overrule the impact of blastoid morphology [4–6]. The transcription regulator SOX11 is expressed in approximately 95% of MCL with nodal presentation [4, 6] including cyclin D1 negative cases [7, 8]. A small proportion of MCL is characterized by non-nodal, leukemic presentation with an indolent disease course and these cases frequently lack expression of SOX11. However, SOX11 negative nodal MCL with *TP53* aberrations are associated with shorter OS in several independent studies [4, 6, 9, 10]. The frequently used clinical prognosticator for MCL is the MCL International Prognostic Index (MIPI), which is based on age, LDH, WBC and ECOG performance status [11]. Since high tumor cell proliferation is associated with impaired survival, the MIPI has been refined to include Ki-67 expression in MIPIb [11].

The current standard treatment for younger MCL patients is rituximab combined with anthracycline- and cytarabine-based regimens followed by consolidating high-dose chemotherapy and autologous stem cells transplantation (ASCT) [2, 12, 13]. While this therapy has significantly improved survival, it is not curative [14]. Among factors predicting a poor response to high-dose treatment and ASCT are *TP53* mutations [5] and high expression of the p53 protein [4, 6]. Positive minimal residual disease (MRD) status pre-ASCT predicts shorter progression-free survival (PFS) and OS [15], suggesting that small lymphoma clones persist after therapy and give rise to progression and eventually relapse.

Sterile α motif and HD domain-containing protein 1, SAMHD1 is expressed in most cells, including leukocytes. SAMHD1 features a deoxynucleoside triphosphate (dNTP) triphosphohydrolase activity that limits the availability of endogenous dNTPs (dTTP, dCTP, dGTP and dATP), and high levels of SAMHD1 prevent virus replication [16]. Importantly, SAMHD1 does not have activity only towards endogenous dNTPs but also towards several nucleoside-based antimetabolites, including cytarabine which is intracellularly metabolized to its active triphosphate form, Ara-CTP [17]. Recently, high levels of SAMHD1 in myeloid blasts were found to correlate with low sensitivity to cytarabine in acute myeloid leukemia [18–20]. There is limited information on the prognostic role of SAMHD1 in lymphoid malignancies [21–23] which led us to investigate the tentative prognostic role in cytarabine treated MCL.

SAMHD1 has other functions independent of the role in maintaining dNTP pools. In CLL, SAMHD1 has been shown to localize to DNA-repair foci suggesting a role in DNA damage response [24]. Recent studies in cell lines have demonstrated that SAMHD1 activates the ATR-CHK1 pathway and promotes resection of nascent DNA at stalled replication forks by activating the MRE11 exonuclease [25]. This suggests that SAMHD1 has a role in preventing tumorigenesis. Thus, SAMHD1 has several functions, including regulation of the dNTP pool, regulation of intracellular antimetabolite levels and functions in DNA repair damage.

We here analyzed the expression of SAMHD1 in MCL and whether it correlated to known negative prognostic factors and outcome after cytarabine treatment and high dose chemotherapy in the context of ASCT. Our aim was to investigate whether SAMHD1 protein expression correlates to progression free survival in MCL, in a similar manner as it does in acute myeloid leukemia.

## Materials and methods

### Patients

A population-based cohort of MCL from the Stockholm region (*N* = 150) was identified and used for analyzing SAMHD1 expression in relation to morphological features. The cohort contained all patients who underwent treatment with six alternating cycles of R-Maxi-CHOP and R-Cytarabine followed by BEAM or BEAC and ASCT, according to the Nordic Lymphoma Group protocols MCL2 and MCL3 [12, 26] at the Karolinska University Hospital between 2000 and 2016 and who had evaluable tissues for immunohistochemistry (*N* = 67). This cohort of ASCT patients was used for investigating OS and PFS (ASCT investigation cohort). A validation cohort for survival analysis (ASCT validation cohort) contained other Scandinavian patients treated in the Nordic MCL2 and MCL3 trials with tissue material for IHC analysis available (*N* = 91).

### Ethical permit

The study has been performed in accordance with the Declaration of Helsinki, including informed patient consent, and has been approved by the Ethical Committee in Stockholm 2018/2182–32.

### Antibodies and IHC analysis

All stainings were done on pre-treatment biopsies. Whole tissue sections (Karolinska patients) and 1 mm diameter tissue microarrays (ASCT validation cohort) were investigated for SAMHD1 expression in tumor cells by dual CD20 (Roche; L26) and SAMHD1 (Proteintech; 12,586–1) immunohistochemistry on a Leica Bond ER robot. In short, pre-treatment was done in citrate buffer, pH 6, for 20 min, thereafter slides were incubated with SAMHD1 antibody 1:200 in Envision Flex diluent (Bond) for 30 min followed by DAB staining (Bond). Thereafter, slides were treated with EDTA buffer, pH 9, for 20 min, followed by CD20 antibody 1:100 for 15 min, followed by Phosphatase Refine Red (Bond). The sections were counterstained by hematoxylin for 10 min. In the Karolinska cases IHC for cyclin D1, Ki67, p53 and SOX11 were done as part of the routine diagnostics and these results were reviewed. Evaluation of Ki67 and p53 immunohistochemistry was done as previously described [4]. p53 positivity was defined as >20% strongly positive tumor cells. SOX11 positivity was defined as >1% positive tumor cells [27] and in 62 of the cases the percentage of SOX11 expressing cells was evaluated. SAMHD1 expression in tumor cells (500 cells × 2 counted for each sample, both cores on the TMA evaluated) was assessed independently by 2 pathologists (EL and BS) each assessing 500 cells. The mean value between the two separate evaluations was used. If there was a discrepancy higher than 10% between pathologists the case was again reviewed by both until agreement.

### Analysis of SAMHD1 and SOX11 mRNA levels in flow cytometry sorted MCL cells

Nineteen specimens of MCL vital frozen cells were thawed, stained for immunoglobulin light chains kappa/lambda, CD5, CD19, CD20, CD3 and the RNA from flow cytometry sorted MCL cells (CD19+, CD20+, CD5+, immunoglobulin light chain restricted; purity>98%) was isolated according to the manufacturer’s protocol using the RNeasy Plus mini kit (Qiagen). RNA quantification was done using the Nanodrop ND-1000 spectrophotometer (Saveen Werner). Complementary DNA (cDNA) was synthesized using the Omniscript Reverse Transcription (RT) kit (Qiagen) according to the manufacturer’s protocol. RNaseOut and Oligo-dT primers were purchased from Invitrogen. SAMHD1 mRNA levels were assessed by Real-Time qPCR using Platinum SYBR Green qPCR Supermix-UDG (Invitrogen) according to the manufacturer’s protocol. Primer sequences were as follows: SAMHD1 forward: 5-AGCGATTGGTTCAAATCCAC-3, reverse: 5-TCGATTGTGTGAAGCTCCTG-3; SOX11 forward 5′-CATGTAGACTAATGCAGCCATTGG-3′, reverse 5′-CACGGAGCACGTGTCAATTG-3′; ACTB forward: 5`-AAAGACCTGTACGCCAACACA-3`, reverse: 5`-AGTACTTGCGCTCAGGAGGA-3`. qPCR reactions were performed using the C1000 Thermal cycler (Bio-Rad). Denaturing was performed at 95 °C for 2 min, followed by 40 cycles at 95 °C for 15 s and 57 °C for 30 s. Cycle threshold (Ct) values of transcripts were quantified using the CFX manager software (BioRad) and ∆Ct values were determined using ACTB as reference. SOX11 and SAMHD1 mRNA in MCL cells were calculated in relation to the SOX11 and SAMHD1 mRNA levels in MCL cell line Granta519 (single cDNA preparation) (∆∆Ct) which was then used to calculate the relative fold increase (RFI).

### Vpx-mediated depletion of SAMHD1

SAMHD1 was depleted in MCL cell lines (Granta-519, JeKo-1 and JVM-2) by means of inactivated virus-like particles (VLPs), including Vpx that targets SAMHD1 protein for ubiquitin-mediated proteolysis within three hours. One million cells were treated with 1 ul of either Vpx, or Vpx-deficient virus-like particles (dX) as a negative control, at conditions of 37 °C and 5% CO_2_ for 72 h and the efficiency of SAMHD1 depletion was confirmed by western blotting (as described below). The preparation and packing of the particles are described in the study of Herold et al. [18] and the relevant references therein. SAMHD1 depletion by Vpx was conducted on 3–5 independent biological replicates.

### Downregulation of SOX11 and SAMHD1 by siRNA

The Granta519, JeKo1 and JVM-2 MCL cell lines were obtained from DSMZ, the German Collection of Microorganisms and Cell Cultures. Granta519 was used to assess the effects of SOX11 and SAMHD1 downregulation by siRNA. The cells were cultured in RPMI 1640 GlutaMAX medium (Gibco), supplemented with 50 μg/ml of gentamicin and 10% fetal bovine serum (Gibco) under the conditions of 5% CO_2_ at 37 °C. Cells were transfected using commercial gene-specific siRNAs (SOX11: s13312; SAMHD1: s24791; 1 μM; Ambion) or negative siRNA, by electroporation method using the AMAXA machine (program X-01) and the Nucleofector kit C (Lonza). After electroporation, the cells were kept in 10% FBS-RPMI-GlutaMAX culture medium, at 37 °C, 5% CO_2_ for 24 h. Cells were counted using NC-200 Automated Cell Counter, NucleoCounter and harvested, washed with PBS and pelleted.

### Western blotting

Proteins were extracted using RIPA buffer (Sigma), supplemented with protease (1:1000, Sigma) inhibitor cocktails, after 30 min incubation on ice. Protein concentrations were measured using BCA assay, with BSA for the standard curve, and 50 μg of protein were resolved on 12% NuPAGE gels (Invitrogen) and transferred using a semi-dry transfer system onto PVDF membranes (Millipore). Non-specific binding sites were blocked with 10% milk TBS-T solution for 1 h at room temperature, then probed overnight at 4 °C with the respective primary antibodies: anti-SAMHD1 (Abcam), anti-SOX11 (Sigma), or anti-Cyclin D1 1:1000 in 5% milk or 5% BSA in TBS-T. Membranes were then washed in TBS-T and probed with secondary antibodies (HRP-conjugated anti-rabbit or anti-mouse; GE Healthcare). Blots were developed using Supersignal West Pico (Pierce) and visualized using LiCor machine. For re-probing of membranes with anti-actin (Sigma) or anti-GAPDH (Cell Signaling) (1:5000 in 5% milk in TBS-T, Sigma), HRP was blocked using the SG substrate kit (Vector Labs). Analysis was done using Fiji-ImageJ software.

### Statistical analysis

Survival times were calculated from the date of commencing therapy (ASCT investigation cohort) or inclusion to trial (ASCT validation cohort) to the date of progression (for progression-free survival [PFS]) or to death for overall survival [OS]). Because our hypothesis was that high SAMHD1 might predict early relapse after cytarabine-based treatment, PFS was chosen as the primary endpoint. Patients were censored at last follow-up. The median follow-up times in surviving patients were in the ASCT investigation cohort 7.3 years (range, 3.5–18.5) and 8.7 years (range, 6.4–14.5) in the ASCT validation cohort. Relationships between independent variables were investigated using Fisher’s exact, Wilcoxon’s, and Spearman’s tests, depending on the nature of the variables. PFS and OS analyses were performed with Kaplan-Meier curves and the log-rank test. Multivariate analyses were conducted using forward stepwise Cox regression; the proportional hazards assumption was checked with graphs based on Schoenfeld residuals. All *P* values are two-tailed and calculated using Stata 14.2 (StataCorp, College Station, TX, USA). *P* < 0.05 was considered significant. Correlation analysis between percentage of SAMHD1+ CD20+ cells by IHC and SAMHD1 mRNA levels in sorted MCL cases and the Spearman correlation between SAMHD1 mRNA and SOX11 mRNA in sorted MCL cells was done using OriginPro 8.

Comparing the expression of Cyclin D1 and SOX11 between dX- and Vpx-treated cells was performed using student t-test (unpaired, two-tailed with Welch correction). The data were represented as mean ± SD and *P* value was set at a cut-off <0.05.

## Results

### SAMHD1 protein expression is variable in MCL and correlates with SAMHD1 mRNA levels

**Fig. 1:**
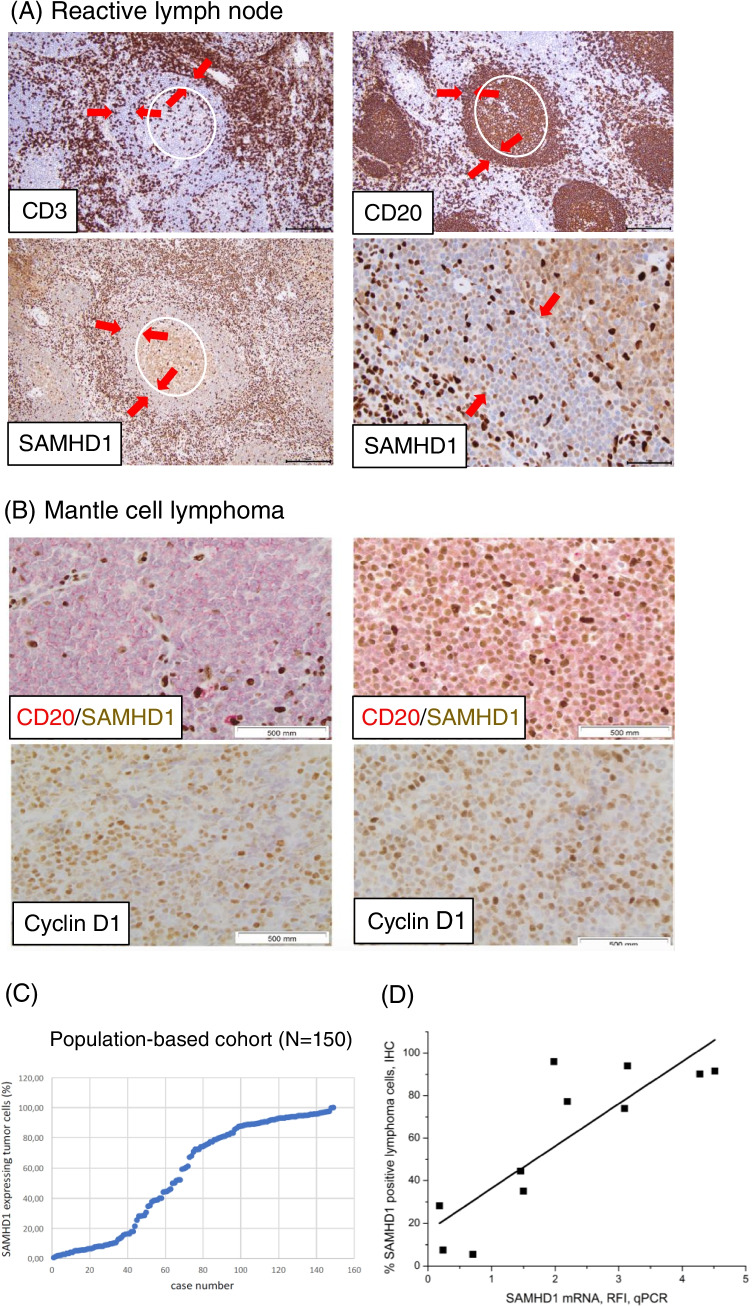
**SAMHD1 expression in reactive lymph node and MCL.** **A**: Reactive lymph node stained for CD3 and for CD20 (upper panels) and for SAMHD1 (lower panels). The germinal center is encircled in white and the mantle zone is indicated by the red arrows. **B**: MCL stained for CD20 in red and SAMHD1 in brown. In MCL SAMHD1 expression is variable with only 2% positive cells in one case (left) and 92% positive cells in another case (right). For comparison cyclin D1 staining of these cases are shown below. **C**: Distribution of SAMHD1 positivity in MCL. MCL cases from the population-based cohort (*N* = 150) are shown on the x-axis and percentage of SAMHD1 positivity on the y-axis. **D**: SAMHD1 mRNA expression in highly enriched MCL cells (kappa/lambda, CD5, CD19, CD20, CD3; purity>98%) correlate to SAMHD1 protein expression by immunohistochemistry in the corresponding biopsies (stained for CD20 and SAMHD1 and the fraction of SAMHD1 expressing CD20 positive cells was evaluated). The variable SAMHD1 protein expression in MCL was confirmed at the mRNA level (Pearson correlation coefficient 0.85, *P* = 0.0009)

We first analyzed SAMHD1 protein expression in reactive lymphoid tissue. SAMHD1 evaluation in non-malignant tonsil or lymph node tissues showed weak to moderate nuclear expression in B cells in mantle zones and germinal centers and strong expression in T cells and macrophages (Fig. 1A). The frequency of SAMHD1 expression in mantle-zone B cells was 30%. Dual staining for CD20 and SAMHD1 in MCL revealed a high variability in the frequency of SAMHD1 positive cells in MCL, and that the intensity of SAMHD1 positivity in MCL nuclei was, with the exception of a few cases, lower than in the lymphoma infiltrating reactive T cells and macrophages (Fig. 1B). SAMHD1 expression in MCL, determined as the proportion of CD20+ cells also expressing SAMHD1, showed a wide range of expression in the Karolinska population-based cohort with a median of 69% (range 0.4–100%) (Table 1, Fig. 1C). Due to variable expression of SAMHD1 in the biopsies we validated our immunohistochemical method for estimating the SAMHD1 levels. Malignant cells from 11 MCL cases were sorted from which mRNA was extracted and *SAMHD1* levels were analyzed by qPCR. Parallel to that, the corresponding diagnostic tissues were stained for SAMHD1. The variable SAMHD1 protein expression in MCL was confirmed at the mRNA level (Pearson correlation coefficient 0.85, *P* = 0.0009; Fig. 1D), suggesting that *SAMHD1* mRNA quantitatively translates to SAMHD1 protein in sorted MCL cells.

**Table 1:** Characteristics of the Stockholm population-based cohort

Factor	Category	Median (min-max)	N	Percent
Age (years)		65 (32–92)	150	
	<50		17	11%
	50–59		28	19%
	60–64		28	19%
	65–69		28	19%
	>70		49	33%
Morphology			148	
	Classical		119	80%
	Blastoid or pleomorphic		29	20%
Ki-67 expression, %	21.5 (1–95)		145	
	<30		89	61%
	≥30		56	39%
SAMHD1, %		69 (0.4–100)	150	
	<10		32	21%
	≥10–50		33	22%
	≥50–90		46	31%
	≥90–100		39	26%
p53 expression >20%			146	
	Negative		126	86%
	Positive		20	14%

### SAMHD1 expression in relation to morphological features

**Fig. 2:**
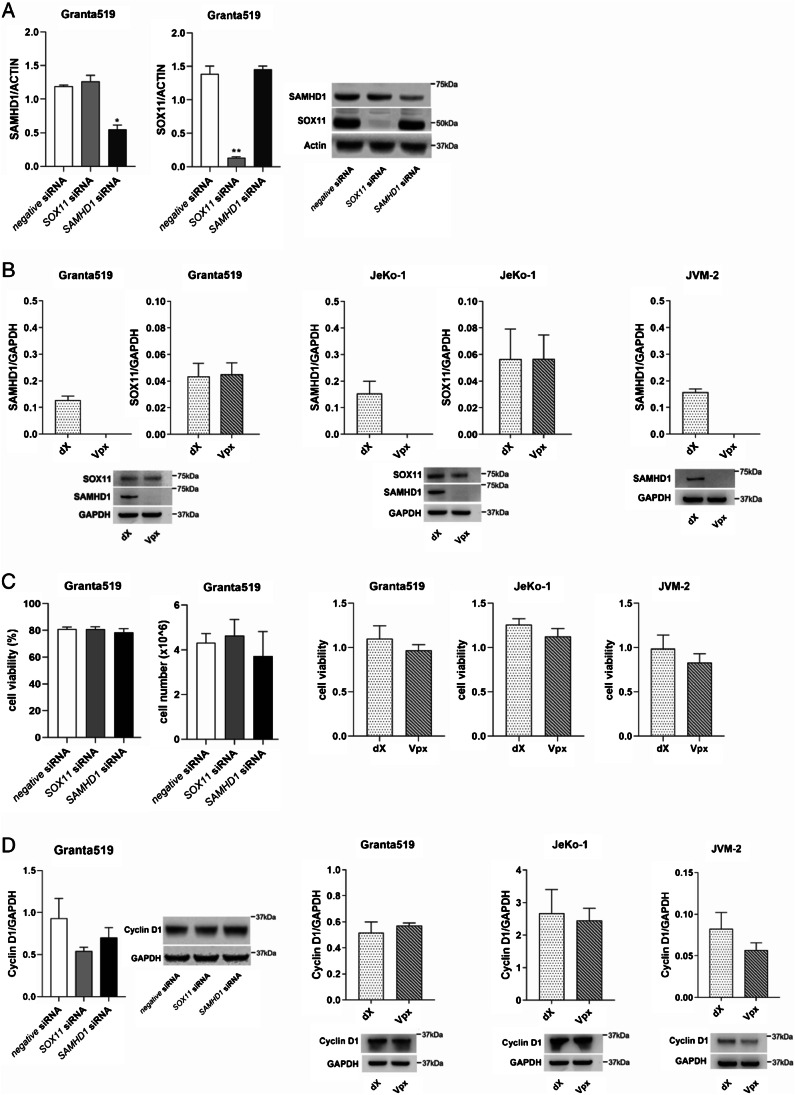
**Protein expression of SAMHD1 and SOX11 after gene silencing of** ***SOX11*** **or** ***SAMHD1*.** **A**: The MCL cell line Granta519 was transfected with specific siRNA targeting *SOX11* or *SAMHD1*, and protein levels were measured 24 h post siRNA by western blot, using beta-actin as loading control. **B**: SAMHD1 was depleted in Granta519, JeKo-1 and JVM-2 cell lines using Vpx treatment for 72 h. Graphs in A and B are averages of three independent replicates, error bars represent standard error of the mean (SEM), *t*-test *p < 0.05, **p < 0.01, and a representative blot for each is shown. **C**: Neither cell number nor cell viability was significantly affected by down-regulation of SOX11 or SAMHD1, or SAMHD1 depletion. **D**: Cyclin D1 protein expression was assessed by western blot after downregulation or depletion of SOX11 or SAMHD1, graphs show averages of 3–5 independent replicates, error bars represent standard error of the mean (SEM), *t*-test was applied, and a representative blot for each experiment is shown

The population-based cohort (*N* = 150) (Table 1) was used for evaluation of possible correlation to morphological characteristics (Table 2). In this cohort, the cases with blastoid/pleomorphic morphology had significantly higher SAMHD1 expression (median 84.9% as compared to 51.9% in cases with classical morphology, Mann-Whitney, *P* = 0.028). SAMHD1 expression was positively correlated to high proliferation (Ki-67 ≥ 30%) (median 80.1% SAMHD1 positive cells as compared to 55.4% in cases with Ki-67 < 30%, Mann-Whitney, *P* = 0.016) but no significant correlation to p53 or SOX11 negativity (Table 2). However, among SOX11 positive cases there was a moderate positive correlation between percentage of SOX11 positive cells and SAMHD1 expression by IHC (*N* = 62, Spearman correlation coefficient 0.27, *P* = 0.036). Further, analysis of mRNA levels of SOX11 and SAMHD1 in sorted MCL cells (*N* = 19) showed a trend for correlation (Spearman correlation coefficient 0.45, *P* = 0.053). However, downregulation of SOX11 by siRNA in Granta519 MCL cell line did not affect the expression of SAMHD1, neither did downregulation of SAMHD1 by siRNA change the expression of SOX11 suggesting that the correlation is not due to a mutual regulation at the gene expression level (Fig. 2A). To further investigate the effect of SAMHD1 on expression of SOX11 we depleted SAMHD1 in MCL cell lines (Granta-519, JeKo-1 and JVM-2) by means of non-infectious virus-like particles (VLPs), including Vpx that targets SAMHD1 protein for ubiquitin-mediated proteolysis within three hours and dX that serves as a control [18]. Downregulation of SAMHD1 by Vpx did not affect the expression of SOX11 in the SOX11+ cell lines Granta519 and JeKo-1 (Fig. 2B). Genetic downregulation of either SOX11 or SAMHD1 in MCL cell line did not affect cell proliferation, viability or cyclin D1 protein expression (Fig. 2C-D).

**Table 2:** SAMHD1 expression in relation to morphological features in the Stockholm population-based cohort

Factor	Category	N	SAMHD1 expression, %; median (min-max)	*P*
Morphology		148		
	Classical	119	51.9 (1–99.6)	0.028
	Blastoid/pleomorphic	29	84.9 (0.4–100)	
Ki-67 expression		145		
	<30%	89	55.4 (1–96.8)	0.016
	≥30%	56	80.1 (0.4–100)	
p53 expression >20%		146		
	Negative	126	72.2 (1–99.6)	0.8
	Positive	20	55.4 (0.4–100)	
SOX11 expression		145		
	Positive	133	72 (0.4–100)	0.8
	Negative	12	61 (3.4–94.8)

### SAMHD1 expression in patients treated according to the Nordic MCL2 and MCL3 protocols

In the population-based cohort 67/150 patients were treated according to the Nordic MCL2/MCL3 protocols and these 67 patients were used as an investigation cohort for PFS and OS. In addition, 91 patients from the Nordic MCL2/MCL3 trials were used as a validation cohort. The clinical and biological characteristics of the investigation and validation cohorts are presented in Table 3. There was no difference in MIPI and MIPIb between the two datasets (both *P* = 0.5). Fewer cases expressed p53 in the investigation cohort (*P* = 0.015). Patients were older in the Karolinska cohort (Table 3), because that dataset also included patients 65–70 years treated according to, but outside, the clinical trials, which had an age limit of 65 years. The 5-year PFS was 65% and 61% in the investigation and validation cohort, respectively (overall, 63%).

**Table 3:** Characteristics of the investigation ASCT and validation ASCT cohorts

	**Investigation cohort (N = 67)**				**Validation cohort (N = 91)**			
**Factor**	**Category**		**N**	**Percent**		**N**	**Percent**	P
Age, years; median (min-max)		61 (32–69)			57 (37–65)			0.005
	<50		13	19%		18	20%	
	50–59		19	28%		42	46%	
	60–64		18	27%		25	27%	
	65–69		17	25%		6	7%	
MIPI; median (min-max)		5.7 (4.7–8.3)			5.7 (4.7–8.7)			0.44
	<5.7		32	48%		51	56%	
	≥5.7–6.5		22	33%		24	26%	
	≥6.5		12	18%		16	18%	
Morphology								0.21
	Classical		57	86%		71	78%	
	Blastoid/pleomorphic		9	14%		20	22%	
Ki-67, %; median (min-max)		21.5 (1–95)			20 (1–91)			
	<20		27	43%		35	41%	
	≥20–30		12	19%		17	20%	
	≥30–50		13	21%		20	24%	
	≥50–70		6	10%		7	8%	
	≥70–95		5	8%		6	7%	
MIPIb; median (min-max)		6.3 (5.2–9.5)			6.1 (4.9–8.8)			0.98
	<5.7		10	15%		15	18%	
	≥5.7–6.5		34	54%		44	52%	
	≥6.5		18	31%		26	31%	
SAMHD1, %; median (min-max)		60 (1–100)			63 (2–100)			0.87
	<10		15	22%		20	22%	
	≥10–50		13	19%		19	21%	
	≥50–90		23	34%		30	33%	
	≥90–100		16	24%		22	24%	
p53 expression >20%								0.015
	Negative		62	97%		37	82%	
	Positive		2	3%		8	18%	

### Survival analysis

**Fig. 3:**
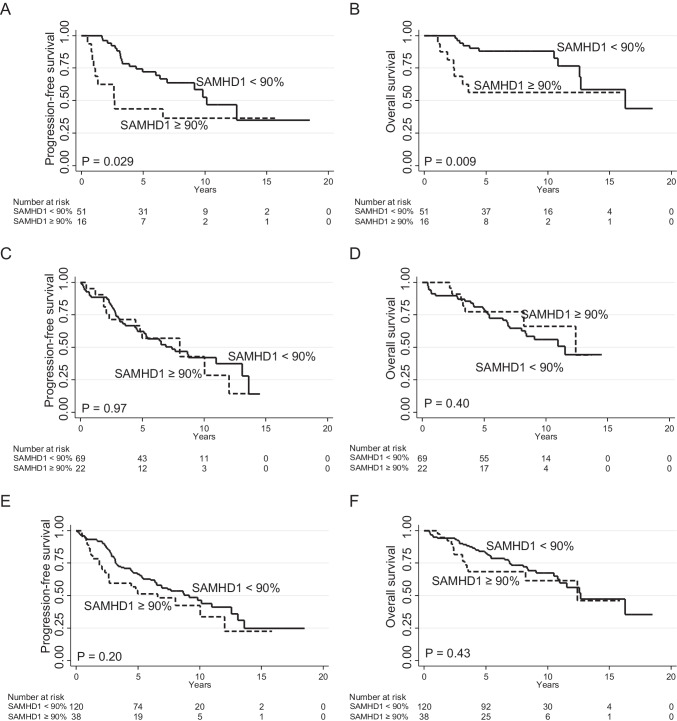
**Long-term outcome according to SAMHD1 expression.** (**A**) Progression-free and (**B**) overall survival in the ASCT investigation cohort (*N* = 67). (**C**) Progression-free and (**D**) overall survival in the validation cohort (*N* = 91). (**E**) Progression-free and (**F**) overall survival in the combined cohort (*N* = 158). *P* values from the log-rank test

SAMHD1 expression was first analyzed in relation to PFS in the ASCT investigation cohort. Low frequency (<10% or < 25%) of SAMHD1+ mantle cell lymphoma cells did not predict PFS, nor did the median (60%) or a 75% cutoff (all *P* > 0.5) (Supplementary Fig. S1). However, patients with ≥90% SAMHD1 positive lymphoma cells had shorter PFS (Fig. 3A) and OS (Fig. 3B). In the investigation cohort, there were 16/67 (24%) patients with ≥90% SAMHD1 positive lymphoma cells, who showed short PFS (at 5 years, 44% compared with 72%). Similarly, patients with blastoid/pleomorphic phenotype showed short PFS compared with classical MCL (at 5 years, 44% vs 69%).

In bivariate analysis for PFS, SAMHD1 but not morphological subtype was independent and morphological subtype was therefore not further analyzed. As shown in Table 2, there was also an association between SAMHD1 and Ki67. However, both factors were independent in a subsequent bivariate analysis, and Ki67 was retained for further adjustment as part of the MIPIb. After multivariate adjustment for the MIPIb, SAMHD1 ≥ 90% remained significant for OS and PFS in the investigation cohort (Table 4).

**Table 4:** Multivariate analyses

Cohort	Analysis	Factor	HR	95% CI	P
Investigation					
	PFS				
		SAMHD1 ≥ 90%	3.1	1.4–6.8	0.005
		MIPIb 5.7–6.5	3.3	0.8–14.7	
		MIPIb ≥6.5	3.7	0.8–17.2	0.24
	OS				
		SAMHD1 ≥ 90%	4.5	1.6–12.7	0.005
		MIPIb 5.7–6.5	3.4	0.3–28.3	
		MIPIb ≥6.5	4.8	0.5–42.2	0.36
Validation					
	PFS				
		SAMHD1 ≥ 90%	0.7	0.3–1.4	0.32
		MIPIb 5.7–6.5	1.1	0.5–2.6	
		MIPIb ≥6.5	3.7	1.5–8.9	0.0003
	OS				
		SAMHD1 ≥ 90%	0.6	0.2–1.4	0.22
		MIPIb 5.7–6.5	1.1	0.3–3.3	
		MIPIb ≥6.5	4.8	1.6–14.3	0.0001
Combined					
	PFS				
		SAMHD1 ≥ 90%	1.3	0.8–2.1	0.37
		MIPIb 5.7–6.5	1.5	0.7–3.1	
		MIPIb ≥6.5	3.1	1.5–6.6	0.002
	OS				
		SAMHD1 ≥ 90%	1.2	0.6–2.3	0.53
		MIPIb 5.7–6.5	1.3	0.5–3.5	
		MIPIb ≥6.5	3.8	1.4–10.1	0.0005

Abbreviations: PFS, progression-free survival; OS, overall survival, MIPIb, Mantle Cell Lymphoma International Prognostic Index (biological); HR, hazard ratio; CI, confidence interval

To validate the importance of the selected cutoff point of ≥90% SAMHD1 positive lymphoma cells, we obtained samples and clinical data from the MCL2 and MCL3 trials (ASCT validation cohort, *N* = 91). Twenty-two out of 91 (24%) patients had ≥90% SAMHD1 positive lymphoma cells. In the validation cohort, SAMHD1 ≥ 90% did not correlate with PFS or OS (Fig. 3C-D) and neither were there any significant correlation to SAMHD1 ≥ 90% in the combined cohorts regarding PFS or OS (Fig. 3E-F).

## Discussion

In this study, we have investigated the expression of SAMHD1 in MCL as compared to non-malignant mantle zone B cells. The expression in normal mantle zone was approximately 30% positive cells. In MCL, the expression pattern was bimodal and one third of cases had fewer than 20% positive cells and half of the cases had >50% SAMHD1 positive cells. We here report that MCL with high frequency of SAMHD1 expressing cells were enriched for cases with blastoid/pleomorphic morphology and high proliferation. We found a moderate correlation between SAMHD1 and SOX11 protein expression in SOX11 positive MCL, and therefore we investigated, using MCL cell lines, whether downregulation of one of these genes would influence expression levels of the other one. As a result, we did not find that SAMHD1 and SOX11 levels mutually regulate protein expression levels. Since SAMHD1 limits the efficacy of cytarabine by hydrolyzing the active metabolite, Ara-CTP, it could be hypothesized that cases with high frequency of SAMHD1 expressing cells are less sensitive to this essential therapeutic component, similar to findings in acute myeloid leukemia [10, 18–20]. Indeed, high SAMHD1 expression in B cell lymphoma cell lines confer resistance to cytarabine in vitro [23]. Cytarabine is however not enough as a single chemotherapy in high risk MCL [28] and the combination treatment given in the MCL2 and MCL3 protocols could explain the lack of significant association between SAMHD1 expression and PFS or OS as here reported by us and recently also by others [23].

SAMHD1 is also considered to have tumor suppressor activity (reviewed in [29]). A recent publication reports a mutation frequency of 7.1% in MCL without a clear correlation to protein expression [3, 23]. Nadeu et al. identified SAMHD1 as one of the recurrent MCL drivers of conventional MCL (mutations or deletions, 10% frequency) [30]. In CLL, mutations were found in 3% pre-treatment and in 11% of refractory/relapsed cases and were associated to lower SAMHD1 protein expression and resistance to agents that induce DNA double strand breaks such as etoposide [24]. Etoposide is part of the MCL high-dose chemotherapy regimen (BEAM and BEAC) administrated prior to the autologous stem cell support [12]. It could therefore be hypothesized that low expression of SAMHD1 in MCL would be associated with resistance to the etoposide component resulting in impaired survival. Our analysis did however not reveal a survival difference in the MCL with low frequency of SAMHD1 expressing cells.

In conclusion, we here report a wide variability in SAMHD1 expression in MCL and a positive correlation to known biological adverse factors such as high tumor cell proliferation and blastoid/pleomorphic subtypes. However, neither low or high SAMHD1 expression correlated to OS or PFS in patients treated according to the Nordic MCL2 or MCL3 protocols.

## Supplementary Information

Below is the link to the electronic supplementary material.

Supplementary file1 (43.4 KB)

## References

[1] Weisenburger DD, Armitage JO (1996). Mantle cell lymphoma-- an entity comes of age. Blood..

[2] Herrmann A, Hoster E, Zwingers T, Brittinger G, Engelhard M, Meusers P (2009). Improvement of overall survival in advanced stage mantle cell lymphoma. J Clin Oncol.

[3] Maddocks K (2018). Update on mantle cell lymphoma. Blood..

[4] Nygren L, Baumgartner Wennerholm S, Klimkowska M, Christensson B, Kimby E, Sander B (2012). Prognostic role of SOX11 in a population-based cohort of mantle cell lymphoma. Blood..

[5] Eskelund CW, Dahl C, Hansen JW, Westman M, Kolstad A, Pedersen LB (2017). TP53 mutations identify younger mantle cell lymphoma patients who do not benefit from intensive chemoimmunotherapy. Blood..

[6] Aukema SM, Hoster E, Rosenwald A, Canoni D, Delfau-Larue MH, Rymkiewicz G (2018). Expression of TP53 is associated with the outcome of MCL independent of MIPI and Ki-67 in trials of the European MCL network. Blood..

[7] Ek S, Dictor M, Jerkeman M, Jirstrom K, Borrebaeck CA (2008). Nuclear expression of the non B-cell lineage Sox11 transcription factor identifies mantle cell lymphoma. Blood..

[8] Mozos A, Royo C, Hartmann E, De Jong D, Baro C, Valera A (2009). SOX11 expression is highly specific for mantle cell lymphoma and identifies the cyclin D1-negative subtype. Haematologica..

[9] Nordstrom L, Sernbo S, Eden P, Gronbaek K, Kolstad A, Raty R (2014). SOX11 and TP53 add prognostic information to MIPI in a homogenously treated cohort of mantle cell lymphoma--a Nordic lymphoma group study. Br J Haematol.

[10] Obr A, Prochazka V, Jirkuvova A, Urbankova H, Kriegova E, Schneiderova P (2018). TP53 mutation and complex karyotype portends a dismal prognosis in patients with mantle cell lymphoma. Clin Lymphoma Myeloma Leuk.

[11] Hoster E, Dreyling M, Klapper W, Gisselbrecht C, van Hoof A, Kluin-Nelemans HC (2008). A new prognostic index (MIPI) for patients with advanced-stage mantle cell lymphoma. Blood..

[12] Geisler CH, Kolstad A, Laurell A, Andersen NS, Pedersen LB, Jerkeman M (2008). Long-term progression-free survival of mantle cell lymphoma after intensive front-line immunochemotherapy with in vivo-purged stem cell rescue: a nonrandomized phase 2 multicenter study by the Nordic lymphoma group. Blood..

[13] Hermine O, Hoster E, Walewski J, Bosly A, Stilgenbauer S, Thieblemont C (2016). Addition of high-dose cytarabine to immunochemotherapy before autologous stem-cell transplantation in patients aged 65 years or younger with mantle cell lymphoma (MCL younger): a randomised, open-label, phase 3 trial of the European mantle cell lymphoma network. Lancet..

[14] Eskelund CW, Kolstad A, Jerkeman M, Raty R, Laurell A, Eloranta S (2016). 15-year follow-up of the second Nordic mantle cell lymphoma trial (MCL2): prolonged remissions without survival plateau. Br J Haematol.

[15] Kolstad A, Pedersen LB, Eskelund CW, Husby S, Gronbaek K, Jerkeman M (2017). Molecular monitoring after autologous stem cell transplantation and preemptive rituximab treatment of molecular relapse; results from the Nordic mantle cell lymphoma studies (MCL2 and MCL3) with median follow-up of 8.5 years. Biol Blood Marrow Transplant.

[16] Baldauf HM, Pan X, Erikson E, Schmidt S, Daddacha W, Burggraf M (2012). SAMHD1 restricts HIV-1 infection in resting CD4(+) T cells. Nat Med.

[17] Herold N, Rudd SG, Sanjiv K, Kutzner J, Bladh J, Paulin CBJ (2017). SAMHD1 protects cancer cells from various nucleoside-based antimetabolites. Cell Cycle.

[18] Herold N, Rudd SG, Ljungblad L, Sanjiv K, Myrberg IH, Paulin CB (2017). Targeting SAMHD1 with the Vpx protein to improve cytarabine therapy for hematological malignancies. Nat Med.

[19] Schneider C, Oellerich T, Baldauf HM, Schwarz SM, Thomas D, Flick R (2017). SAMHD1 is a biomarker for cytarabine response and a therapeutic target in acute myeloid leukemia. Nat Med.

[20] Rassidakis GZ, Herold N, Myrberg IH, Tsesmetzis N, Rudd SG, Henter JI (2018). Low-level expression of SAMHD1 in acute myeloid leukemia (AML) blasts correlates with improved outcome upon consolidation chemotherapy with high-dose cytarabine-based regimens. Blood Cancer J.

[21] Buhler MM, Lu J, Scheinost S, Nadeu F, Roos-Weil D, Hensel M (2021). SAMHD1 mutations in mantle cell lymphoma are recurrent and confer in vitro resistance to nucleoside analogues. Leuk Res.

[22] Xagoraris I, Vassilakopoulos TP, Drakos E, Angelopoulou MK, Panitsas F, Herold N (2021). Expression of the novel tumour suppressor sterile alpha motif and HD domain-containing protein 1 is an independent adverse prognostic factor in classical Hodgkin lymphoma. Br J Haematol.

[23] Roider T, Wang X, Huttl K, Muller-Tidow C, Klapper W, Rosenwald A (2021). The impact of SAMHD1 expression and mutation status in mantle cell lymphoma: an analysis of the MCL younger and elderly trial. Int J Cancer.

[24] Clifford R, Louis T, Robbe P, Ackroyd S, Burns A, Timbs AT (2014). SAMHD1 is mutated recurrently in chronic lymphocytic leukemia and is involved in response to DNA damage. Blood..

[25] Coquel F, Silva MJ, Techer H, Zadorozhny K, Sharma S, Nieminuszczy J (2018). SAMHD1 acts at stalled replication forks to prevent interferon induction. Nature..

[26] Kolstad A, Laurell A, Jerkeman M, Gronbaek K, Elonen E, Raty R (2014). Nordic MCL3 study: 90Y-ibritumomab-tiuxetan added to BEAM/C in non-CR patients before transplant in mantle cell lymphoma. Blood..

[27] Lord M, Wasik AM, Christensson B, Sander B (2015). The utility of mRNA analysis in defining SOX11 expression levels in mantle cell lymphoma and reactive lymph nodes. Haematologica..

[28] Laurell A, Kolstad A, Jerkeman M, Raty R, Geisler CH (2014). High dose cytarabine with rituximab is not enough in first-line treatment of mantle cell lymphoma with high proliferation: early closure of the Nordic lymphoma group mantle cell lymphoma 5 trial. Leuk Lymphoma.

[29] Herold N, Rudd SG, Sanjiv K, Kutzner J, Myrberg IH, Paulin CBJ (2017). With me or against me: tumor suppressor and drug resistance activities of SAMHD1. Exp Hematol.

[30] Nadeu F, Martin-Garcia D, Clot G, Diaz-Navarro A, Duran-Ferrer M, Navarro A (2020). Genomic and epigenomic insights into the origin, pathogenesis, and clinical behavior of mantle cell lymphoma subtypes. Blood..

