# Supplementary material for: Clinical and biological impact of SAMHD1 expression in mantle cell lymphoma
Source: Virchows Arch. 2021 Nov 4;480(3):655–66. doi: 10.1007/s00428-021-03228-w (PMC8989861; doi:10.1007/s00428-021-03228-w)
Supplement: Supplementary file 1 — Supplementary file1 (43.4 KB) [file 428_2021_3228_MOESM1_ESM.pptx]

## Slide 1
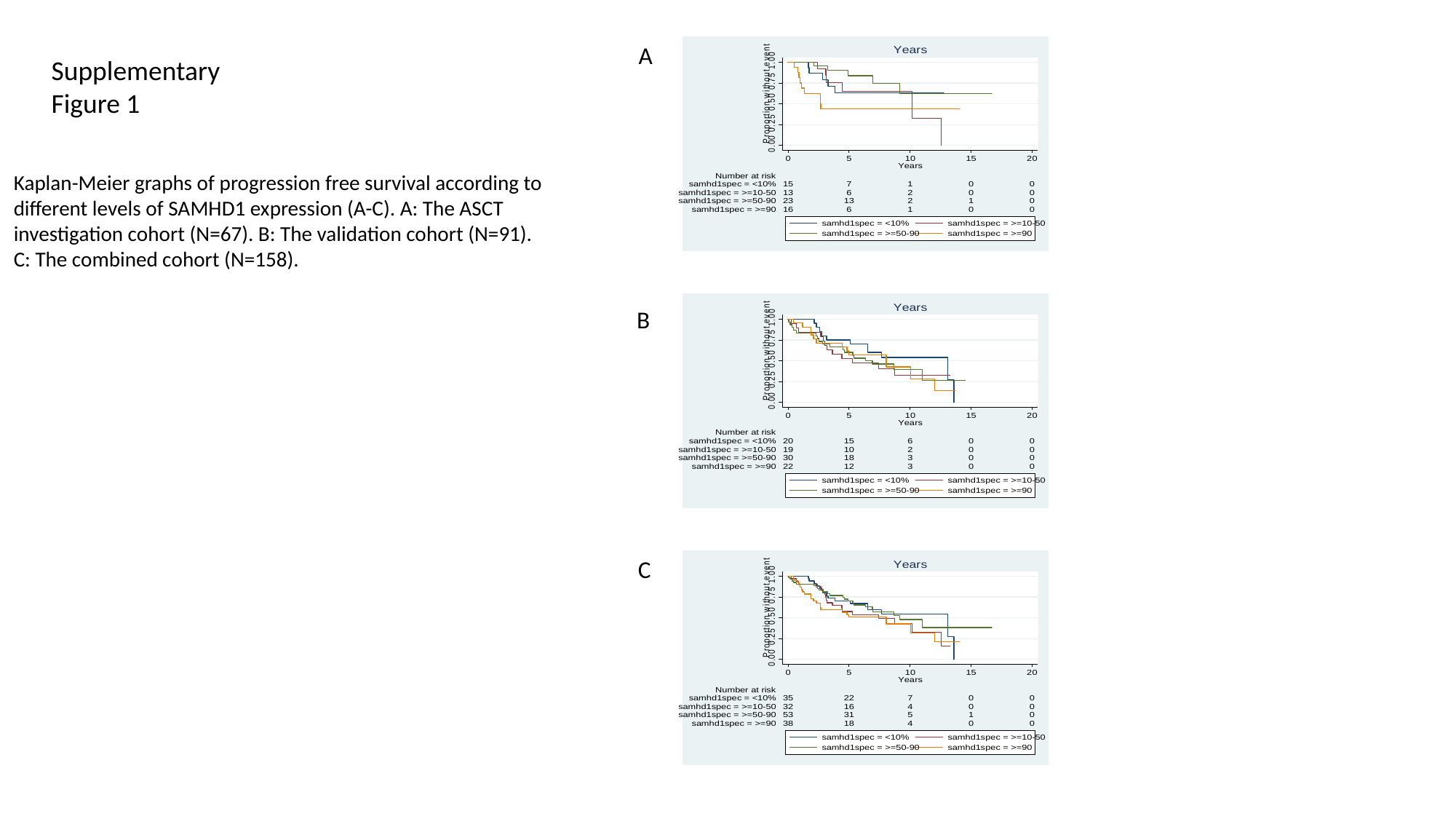

A
B
C
Supplementary
Figure 1
Kaplan-Meier graphs of progression free survival according to
different levels of SAMHD1 expression (A-C). A: The ASCT
investigation cohort (N=67). B: The validation cohort (N=91).
C: The combined cohort (N=158).
